# Basaloid squamous cell carcinoma: two case reports

**DOI:** 10.1186/1757-1626-2-9351

**Published:** 2009-12-18

**Authors:** Pooja Vasudev, Odette Boutross-Tadross, Jasim Radhi

**Affiliations:** 1 Department of Pathology, McMaster University, 1200 Main Street West, Hamilton, Ontario, L8N 3V7, Canada

## Abstract

Basaloid squamous cell carcinoma (BSCC) is a rare and aggressive variant of squamous cell carcinoma (SCC) that occurs preferentially in the upper aerodigestive tract. We present two cases of BSCC, one arising in the conjunctiva and the other arising in a paranasal sinus. Clinical and pathological findings in these two cases, including immunohistochemistry is presented along with brief discussion of literature. To the best of our knowledge, this is the first report of BSCC of the conjunctiva. BSCC of the head and neck should be distinguished from adenoid cystic carcinoma, small cell neuroendocrine carcinoma, basal cell adenocarcinoma, adenosquamous carcinoma, squamous cell carcinoma, spindle cell squamous carcinoma, mucoepidermoid carcinoma, and adenoid cystic carcinoma.

## Introduction

Basaloid squamous cell carcinoma (BSCC) was first described in the upper aerodigestive tract by Wain et al [1] in 1986. BSCC is characterized by nesting, Lobular and trabecular arrangement of small crowded cells with scant cytoplasm, and hyperchromatic nuclei. The lobules of malignant basaloid cells often display peripheral nuclear palisading, high mitotic activity, comedo necrosis, and small cystic spaces filled with mucinous material, making these tumors difficult to differentiate from adenoid cystic carcinoma or from small-cell undifferentiated carcinoma.

The most common sites to be affected are larynx, hypopharynx, tonsils and the base of tongue. Other less frequently affected sites are nose, paranasal sinus, external ear, submandibular region, esophagus, lung, anus, vulva, vagina and the uterine cervix. So far, only 21 cases of BSCC of the nose and paranasal sinuses have been reported in the English literature [2]. To our best knowledge, this tumor has not been reported in the conjunctiva.

## Case Reports

Our first case was a 64-year-old Caucasian Canadian male who presented with an eyelid mass. On examination, it was found to be arising from the palpebral conjunctiva. A biopsy was taken from the tumor and interpreted initially as basal cell carcinoma. This was followed by wider excision following review of pathology at our institution. Past medical history was unremarkable. The second case was a previously healthy 60-year-old female who presented with epistaxis, pain and mass in the right nasal cavity and paranasal sinus. Preoperatively, the mass was found to be arising from the anterior end of right middle turbinate of the nasal cavity. It was clinically thought to be a hemangioma. There was no significant history of smoking or alcohol consumption in either case. There was no evidence of either lymphatic or distant metastasis at the time of diagnosis. The second case showed a low absolute lymphocyte count.

**Figure 1:**
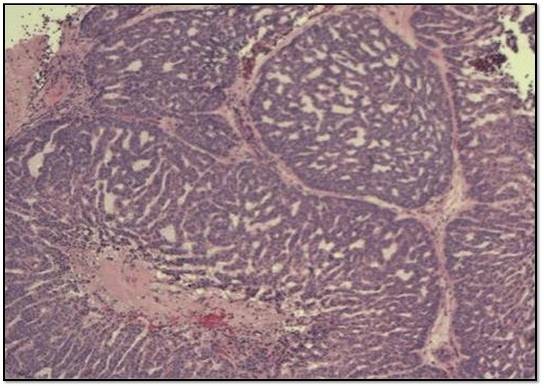
**Microphotograph of BSCC showing islands of tumor cells with peripheral palisading and central necrosis**.

**Figure 2:**
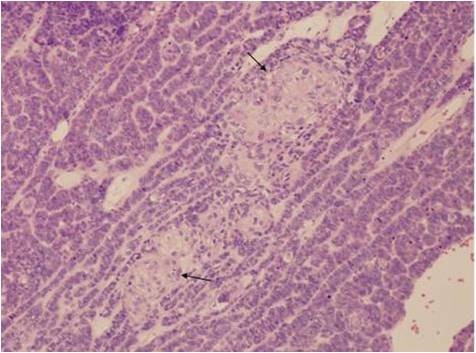
**Microphotograph of BSCC showing squamous cells nesting**.

**Figure 3:**
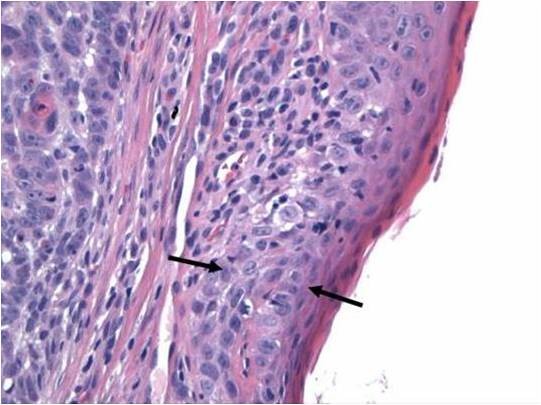
**Bssc with surface epithelial dysplasia**.

**Figure 4:**
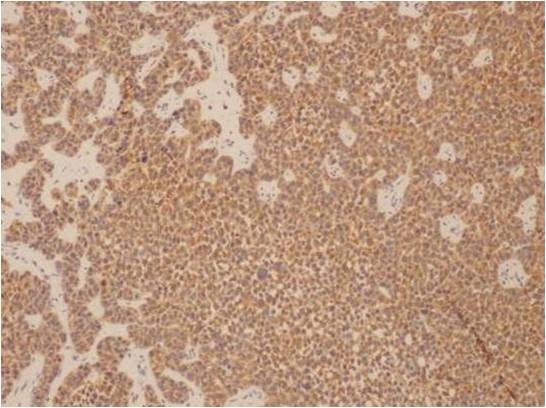
**Microphotograph immunostating of BSCC with .34 BE 12 marker**.

**Figure 5:**
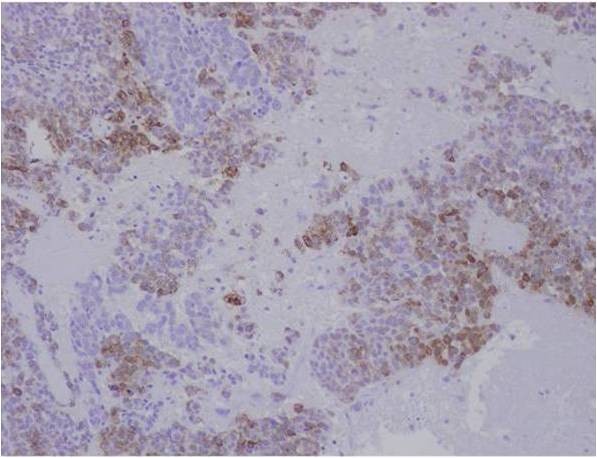
**Microphotograph of immunostating of BSCC with EMA marker**.

Microscopic examination of both cases showed tumour cells arranged in ribbons, trabeculae and islands. There were two types of tumour cells. The predominant cell population was that of basaloid, closely apposed cells with scanty cytoplasm and hyperchromatic nuclei. Nucleoli were variable. Islands of tumour cells showing peripheral palisading and central necrosis were noted in case 1 (Figure 1). There was an abrupt association of these basaloid cells with rare squamous foci (Figure 2). Some multinucleated giant cells containing intracellular keratin were also seen. Many apoptotic cells and mitotic figures, including atypical mitosis were present. Tumour was seen to arise from the surface epithelium which was focally dysplastic (Figure 3). Few rosette-like structures were noted in case 1. Many thin walled blood filled channels as well as areas of hemorrhage were present in case 2. The immnoprofile of these tumours showed diffuse positive staining for squamous epithelial marker 34βE12 (Figure 4) and are negative for other markers for small cell neuroendocrine tumours and myoepithelial markers seen in adenoid cystic carcinoma. In addition these lesions were positive for EMA marker (Figure 5) and negative for BerEP4 and BCL2 which were helpful to differentiate these tumours from BCC. The conjuctival lesion was treated with wider excision and follow up. The patient with the nasal BSCC was treated by surgical excision of the mass together with anterior end of the middle nasal turbinate in October 2007, followed by radiotherapy. At last follow-up, 19 months after surgery, the patient exhibited no evidence of recurrent disease or regional lymph node metastases.

## Discussion

BSCC is a rare variant of squamous cell carcinoma which occurs predominantly in men in their 60 and 70s. There have been some reports of it being associated with tobacco and alcohol abuse [2]. It arises in a variety of anatomic sites, most frequently in the upper aero-digestive tract with strong predilection for the base of the tongue, supraglottic larynx and hypopharynx, but is also found in the anus, thymus and uterine cervix. Clinically, it is an aggressive tumor with high rates of nodal (64%) and distant metastasis (44%). Results of a case-control study by Soriano et al [3] found a 6 times higher risk of distant metastasis compared to usual type of SCC. Some authors, therefore, recommend a chest CT and FDG-PET in all cases to rule out early distant metastasis. Treatment of choice is complete surgical excision supplemented by radiotherapy/adjuvant chemotherapy. Nasal BSCC usually presents as either an obstructive mass or pain. Wieneke et al [4] studied 14 cases of BSCC of the sinonasal tract. All their reported cases demonstrated surface epithelial dysplasia, however there was no evidence of multifocal disease. In addition, to the usual morphological features of BSCC they found neural type rosettes arrangement. Both of our cases corroborate their findings. Due to its biphasic nature, BSCC may not be recognized if the minor squamoid component is overlooked or not sampled, especially in small biopsies. BSCC needs to be differentiated from tumours such as adenoid cystic carcinoma (ACC) [5], as it carries a poorer prognosis and has different management regimes. BSCC may have PAS positive microcystic spaces similar to the characteristic punched out spaces of ACC. However, ACC often has less pleomorphism, mitosis and necrosis than BSCC. There is no dysplasia or continuity of tumor with the surface epithelium in adnexal tumors such as ACC. It is important to distinguish BSCC from small cell neuroendocrine carcinoma (SCNC) because of the difference in treatment for these two tumors. Both tumors may show sheets of small blue cells and rosette like structures (Figure 3). However, SCNC shows characteristic nuclear molding and crushing artifact, and is rarely connected to surface mucosa. Basal cell carcinoma is extremely rare primary conjuctival tumor [6]. However, BCC arising from eyelid skin with direct spread to conjuctival mucosa needs to be considered in the differential diagnosis of the first case. The BCCs are made up of nests of basaloid cells with peripherally palisading cells. The cells have hyperchromatic nuclei and scant cytoplasm. The nests of tumor cells are surrounded by stroma. Retraction spaces can usually be observed between the islands and stroma. Ulcerations may be seen in large tumors, but no dysplasia of the surface epithelium. BSCC is distinguished on the basis of histologic inspection by lobules, nests, and cribriform patterns of basaloid cells that commonly have abrupt foci of squamous differentiation within the nests. Necrosis is typical, taking the form of single cell necrosis and central comedo necrosis. The second major characteristic of the BSCC is the presence of squamous component that includes at least one of following features: adjacent foci of conventional squamous cell carcinoma, dysplasia or carcinoma in situ of the overlying mucosa. In the large majority of cases, the distinction between these two tumours is readily made on the basis of standard H&E morphology. However, application of immunohistochemical markers is useful to differentiate these tumors. BCCs are negative for EMA, and are positive for BCL2 and 2 Ber-EP4. The cells in BSCC are positive for 34βE12 and EMA and focally positive for CEA [7]. The lesion was clinically conjunctival in origin and associated with surface epithelial dysplasia, and pathologically supported by immunohistochical application fulfills the features of BSCC. To our knowledge this represents the first case of BSCC to be reported in the conjunctiva.

## Consent

Written informed consent could not be obtained. The patient's identity remains anonymous, and we would not expect the patients or their families to object to publication.

## Competing interests

The authors declare that they have no competing interests.

## Authors' contributions

PV performed literature search, wrote initial manuscript. OBT and JR provided the histopathological diagnosis and contributed to the writing and editing of the manuscript. All authors read and approved the final manuscript.
